# Chloroplast Genome Sequence of *Arabidopsis thaliana* Accession Landsberg *erecta*, Assembled from Single-Molecule, Real-Time Sequencing Data

**DOI:** 10.1128/genomeA.00975-16

**Published:** 2016-09-22

**Authors:** Kai Bernd Stadermann, Daniela Holtgräwe, Bernd Weisshaar

**Affiliations:** a Chair of Genome Research, Faculty of Biology, Bielefeld University, Bielefeld, Germany; b Bioinformatics Resource Facility, Centre for Biotechnology, Bielefeld University, Bielefeld, Germany

## Abstract

A publicly available data set from Pacific Biosciences was used to create an assembly of the chloroplast genome sequence of the *Arabidopsis thaliana* genotype Landsberg *erecta*. The assembly is solely based on single-molecule, real-time sequencing data and hence provides high resolution of the two inverted repeat regions typically contained in chloroplast genomes.

## GENOME ANNOUNCEMENT

*Arabidopsis thaliana* is the most used model organism in plant genetics. During the last decades, various genotypes of *A. thaliana* have been sequenced using different sequencing technologies (1, 2). With third-generation sequencing technologies entering the market, novel techniques for DNA sequence generation have become available. The L*er*-0 genotype is currently the second most widely used accession of *A. thaliana* behind Columbia Col-0. The latest published L*er*-0 genome assembly used reads created by third-generation sequencing along with reads from other sources to create a high-quality nuclear genome assembly (3). One of the new technologies is single-molecule, real-time (SMRT) sequencing (4) developed by Pacific Biosciences, which is sometimes referred to as “PacBio sequencing”. This sequencing technology allows long read lengths up to the 20- to 60-kb range, allowing single reads to span long repetitive elements or regions. The typical chloroplast genome contains two of these long (~26 kb) repeat regions, with the additional feature that the two regions are inverted repeats (IR) of each other. These IRs are hard to assemble using only short-read data from next-generation sequencing methods.

Recently, we described an approach to assemble a chloroplast genome based on an SMRT sequencing data set originally dedicated to sequencing a nuclear genome (5). We applied this approach to data from a single SMRT cell obtained from a publically available SMRT sequencing data set for *Arabidopsis thaliana* L*er*-0 provided by Pacific Biosciences (6). Sequence reads from sample number SAMN02724977 created by P5-C3 chemistry were used. We followed our protocol for identification of potential chloroplast reads, assembly, alignment, and polishing as described previously (5) with one exception: instead of using the spinach chloroplast genome sequence (7) as a template for read extraction, we used the chloroplast genome sequence of the *Arabidopsis thaliana* Col-0 genotype (8). We extracted 11,016 subreads summing up to 90,383,214 bp. The assembly resulted in a sequence with overlapping ends, and hence we assume that the chloroplast genome sequence is complete. We removed the additional overlap and aligned the sequence to the standard starting position of chloroplast genome sequences. The resulting assembly, designated Ler0_cp_smrt, has a total length of 154,515 bp and differs in 111 positions from the Col-0 chloroplast genome sequence (AP000423.1). We annotated the genome using CpAVAS (9) and identified 123 genes. Of these, 85 genes encode mRNA (i.e., proteins), 8 encode rRNA, and 30 encode tRNA.

### Accession number(s).

The complete sequence of the *Arabidopsis thaliana* L*er*-0 chloroplast genome was deposited in GenBank under accession number KX551970.
